# Exon Shuffling Played a Decisive Role in the Evolution of the Genetic Toolkit for the Multicellular Body Plan of Metazoa

**DOI:** 10.3390/genes12030382

**Published:** 2021-03-08

**Authors:** Laszlo Patthy

**Affiliations:** Institute of Enzymology, Research Centre for Natural Sciences, H-1117 Budapest, Hungary; patthy.laszlo@ttk.mta.hu; Tel.: +36-13826751

**Keywords:** cell-cell adhesion, cell-cell signaling, choanoflagellates, exon shuffling, genetic toolkit, introns, metazoan origins, multicellularity, temporal-to-spatial transition, self-nonself recognition

## Abstract

Division of labor and establishment of the spatial pattern of different cell types of multicellular organisms require cell type-specific transcription factor modules that control cellular phenotypes and proteins that mediate the interactions of cells with other cells. Recent studies indicate that, although constituent protein domains of numerous components of the genetic toolkit of the multicellular body plan of Metazoa were present in the unicellular ancestor of animals, the repertoire of multidomain proteins that are indispensable for the arrangement of distinct body parts in a reproducible manner evolved only in Metazoa. We have shown that the majority of the multidomain proteins involved in cell–cell and cell–matrix interactions of Metazoa have been assembled by exon shuffling, but there is no evidence for a similar role of exon shuffling in the evolution of proteins of metazoan transcription factor modules. A possible explanation for this difference in the intracellular and intercellular toolkits is that evolution of the transcription factor modules preceded the burst of exon shuffling that led to the creation of the proteins controlling spatial patterning in Metazoa. This explanation is in harmony with the temporal-to-spatial transition hypothesis of multicellularity that proposes that cell differentiation may have predated spatial segregation of cell types in animal ancestors.

## 1. Introduction

Transitions to multicellularity occurred independently multiple times, leading to complex organisms such as animals, land plants, fungi, and red, green, and brown algae [1,2,3,4,5].

There are two major mechanisms for unicellular to multicellular transition: aggregation, when separate cells adhere to each other, or clonal development, when multicellularity results from cell divisions without separation of sister cells. It is also important to distinguish multicellular forms with respect to their complexity. The so-called ‘simple’ multicellular forms usually consist of similar cells and all cells are in direct contact with the external environment. On the other hand, ‘complex’ multicellular organisms consist of different cell types that occupy different positions relative to the external environment [3].

The fact that multicellular organisms evolved independently several times indicates that, in general, there must be positive selection for multicellularity. There are numerous reasons why multicellularity could provide selective advantage over unicellularity [6,7,8]. It is widely accepted that an important driving force of multicellularity is that, thanks to their increased size, multicellular forms are less vulnerable to predation than the unicellular forms. The most obvious major benefit of multicellularity, however, is that different cells of the multicellular form have the capacity for the division of labor and the coexistence of different cell types allows the organism to perform a variety of cellular functions simultaneously and with increased efficiency.

Division of labor, however, requires cooperation of different cell types, with concomitant loss of their evolutionary autonomy and the transfer of the criteria of fitness from individual cells to the community of cells that constitute the organism [9]. Maintenance of a stable multicellular state requires ‘ratcheting’ mechanisms that ensure that cell–cell cooperation is favored over cellular autonomy to avoid the danger that individual cells ‘exploit’ the organism, as it occurs in cancer [9]. It has been suggested that division of labor by cells of multicellular organisms has not only the benefits of specialization, but also serves as a ratcheting trait as cells performing a given specialized function must depend on functions provided by other cells [9].

Recent phylogenetic and genomic studies on animals, plants, and algae suggest a similar trajectory for the independent evolution of their complex multicellularity [3,4,5]. All complex multicellular organisms show evidence for a need for cell–cell adhesion, cell–cell communication, differentiation of cell types mediated by networks of regulatory genes, and a developmental program. Despite these similarities, the genes fulfilling the roles of cell–cell adhesion, cell–cell communication, and development are markedly different in the different types of complex multicellular organisms [3,4,5].

## 2. Evolution of Metazoa

In this paper, I will focus on the unicellular-multicellular transition in the Metazoa lineage and the evolution of the genomic toolkit responsible for metazoan type multicellularity.

### 2.1. Unicellular to Multicellular Transition in the Metazoan Lineage

Thanks to studies in the last two decades, there is now a clear consensus that choanoflagellates, a group of marine and freshwater protozoans, are the closest living relatives of animals [10,11,12,13,14]. Comparison of the biology, genomics, transcriptomics, and proteomics of choanoflagellates and animals, thus, permits some insight into the unicellular to multicellular transition of the metazoan ancestor.

Significantly, the life cycles of all choanoflagellates contain a single-celled phase but many species are also capable of forming multicellular colonies. These simple multicellular forms include spherical rosette colonies or linear chain colonies [15,16]. The multicellular forms of choanoflagellates are similar to animals in that they also develop clonally [17,18]. Interestingly, in response to environmental changes, the choanoflagellate *Salpingoeca rosetta* differentiates into several distinct cell types. There are solitary cell types (thecate cells, slow swimmers, fast swimmers, amoeboid forms) and two colonial forms, rosettes and chains [18,19]. Thecate cells are sedentary, they attach to a substrate through a secreted goblet-shaped theca. Choanoflagellates subjected to confinement differentiate from a flagellated form to an amoeboid form by retracting their flagella and activating myosin-based motility [19].

Rosette colonies develop from a subpopulation of slow swimmers, suggesting that molecular differentiation precedes the onset of colony formation [18]. Interestingly, even cells in the same choanoflagellate rosette colony differ from each other in cell morphology, suggesting that they have the capacity for differentiation [7].

A recently described choanoflagellate species, *Choanoeca flexa*, displays even more remarkable signs of transition to more complex multicellularity. Cup-shaped colonies of *C. flexa* rapidly invert their curvature in response to changing light levels allowing alternation between feeding and swimming behavior [20]. This inversion of colonies requires actomyosin-mediated apical contractility, indicating that apical cell contractility evolved before the origin of animal multicellularity.

Recent studies have provided some insight into the similarities and differences of the developmental programs that control the formation of different unicellular and multicellular forms of choanoflagellates and complex multicellular forms of animals.

Although the evolution of cell types is critical for understanding metazoan evolution, relatively little is known about the evolution of the underlying gene regulatory programs. In a recent study, Sebé-Pedrós et al. [21] have used whole-organism single-cell RNA-seq to map cell type-specific transcription in Porifera, Ctenophora, and Placozoa species and have characterized the repertoires of cell types in these earliest-branching animal lineages. The authors have demonstrated that distinct networks of transcription factors and proximal promoters, that constitute cell type-specific transcription factor modules, clearly define the different metazoan cell types of these animals.

Morphologically and behaviorally different cell types of *S. rosetta* also have distinct transcriptional profiles, in harmony with the notion that differentiation of cells and colony formation is also a regulated developmental process, controlled by transcription factor modules [22]. Interestingly, genes shared exclusively by metazoans and choanoflagellates were disproportionately upregulated in choanoflagellate colonies and the single cells from which they develop, suggesting that this gene set may have provided the foundations for the regulation of cell differentiation in Metazoa [22].

The existence of multiple cell types in choanoflagellates suggests that the evolution of cell differentiation predated the evolution of complex multicellularity in the Metazoa lineage. This conclusion is in harmony with the temporal-to-spatial transition hypothesis that assumes that temporally alternating phenotypes of the unicellular ancestor of Metazoa were converted into spatially juxtaposed cell types [14,23,24]. According to this hypothesis, the unicellular to multicellular transition is essentially a transition from temporally regulated to spatiotemporally regulated cell type differentiation. It has been suggested that the different cell types of the multicellular organism could appear simply by selectively shutting down or switching on cell type-specific transcription factor modules, according to a developmental program [14].

Recent discoveries provide evidence for such a scenario. First, unicellular relatives of animals have complex life cycles with different cell types and temporally regulated multicellular states [20,22,25]. Second, the temporally regulated cell type differentiation of unicellular relatives of animals is associated with specific transcriptional profiles, arguing for the existence of distinct, cell type specific transcription factor modules [22,26,27]. Finally, the observation that a significant proportion of the metazoan developmental transcription factors originated in unicellular eukaryotes [28] suggests that the transcription factor modules specifying cell types of Metazoa evolved from transcription modules of their premetazoan ancestors. All these findings suggest that the different cell types of premetazoans, such as choanoflagellates are the result of differentiation processes, consistent with the view that regulated cell differentiation existed before the emergence of animal multicellularity. The first animal cells could transition between multiple states in a way similar to stem cells [29].

An important aspect of the temporal-to-spatial transition hypothesis is that it separates two major preconditions of complex multicellularity: differentiation of cell types and their spatial patterning. This distinction is quite remarkable in view of the fact that although different cell types of choanoflagellates have distinct transcriptional profiles, their colonial forms are not known to undergo significant spatial cell differentiation. Accordingly, the most critical difference between simple and complex multicellularity is that the latter requires the evolution of morphogenetic programs, genetic toolkits that allow the different cell types to co-exist in a well-defined spatial arrangement.

A necessary but not sufficient condition of spatial patterning in multicellular organisms is that cells interact with and adhere to other cells. Simple and complex multicellularity are similar in that both require systems allowing the recognition of and discrimination between self and nonself, between identical and nonidentical cell types [6]. As pointed out by Grice and Degnan [6], in the self–nonself recognition process the cell detects the presence of another cell in its vicinity, recognizes it as self or nonself and then takes some action based on the recognition decision. The importance of discrimination between self and nonself is particularly obvious in the case of multicellular aggregates when nonself cells might threaten the integrity of the multicellular entity [6].

Clearly, the functional requirements of self–nonself recognition is intimately related to cell adhesion processes as both systems require the presence of cell surface proteins that interact specifically to facilitate binding to and communication between cells. The structural requirements of self–nonself recognition and cell adhesion are also similar; both of these processes usually employ transmembrane proteins with large extracellular regions, such as various members of the cadherin, immunoglobulin and selectin cell adhesion families [6].

The ability of cells to recognize identical or different cell types, to adhere preferentially to their own type, however, is even more critical for the morphogenesis of complex multicellular forms as selectivity in cell–cell adhesion has a key role in the organization of tissues. For example, through their homophilic binding interactions, cadherins play a role in cell-sorting mechanisms, conferring adhesion specificities on cells [30,31]. As cells differentiate, they may acquire differential adhesive properties allowing cells to sort out from one another, permitting the establishment of complex three-dimensional patterns of cells in tissues [32,33,34].

It is plausible to assume that in the premetazoan unicellular ancestor, a primordial self–nonself recognition/cell adhesion system could serve to maintain a simple multicellular form consisting of similar cells. This system could protect the multicellular form from ‘invasion’ by nonself cells simply by passive discrimination: cells recognized as self through homophilic binding remain attached, whereas nonself cells are released. It is noteworthy in this respect that cadherin genes are present in choanoflagellates in large numbers, similar to those observed in complex Metazoa [35,36]. It seems, thus, likely that the common ancestor of choanoflagellates and animals had two key features of multicellularity: the existence of different, temporally alternating cell types and adhesive properties that allow cells to adhere to each other. Transition to complex multicellularity in Metazoa, however, required the evolution of a toolkit that allows different cell types to co-exist and that helps arrange them into unique three-dimensional patterns that are optimal for the integration of their distinct functions.

### 2.2. The Genetic Toolkit of the Multicellular Body Plan of Metazoa

As mentioned above, a significant proportion of the metazoan developmental transcription factors were present in the unicellular ancestors of animals, suggesting that the transcription factor modules of Metazoa evolved from transcription regulatory networks of their premetazoan ancestors [28]. There are, however, transcription factors that evolved only at the onset of Metazoa [28]. For example, during metazoan evolution, Homeobox genes increased from a rather simple gene complement to a highly diversified toolkit and this expansion coincided with an increase in domain combinations [37]. Analyses of sponge, cnidarian, placozoan, and choanoflagellate genomes have also revealed that many of the transcription families are metazoan specific, suggesting that the genesis and expansion of these families contributed to the evolution of animal multicellularity [38,39]. The increase in the size of transcription factor families of Metazoa has been shown to correlate with morphological and cell type complexity, suggesting that an increase in their number is closely associated with the appearance of new cell types. The emergence of novel transcription factor genes prior to the separation of modern animal lineages supports the hypothesis that these innovations provided the basis for the evolution of multicellularity and embryogenesis [38,39].

In Metazoa, embryogenesis and pattern formation is initiated by some cytoplasmic determinants that, following cleavage of the egg, are transferred to only some of the cells. Cells that contain the determinant initiate the developmental pathway through the production of extracellular morphogens such as Wnts, hedgehog factors, bone morphogenetic proteins of the Transforming Growth Factor β (TGFβ) family, fibroblast growth factors [40]. Morphogens usually form extracellular concentration gradients, upregulating or downregulating different genes at different concentration thresholds through interaction with their receptors [41]. Interactions of morphogens with their transmembrane receptors trigger cellular responses through signal transduction pathways that connect extracellular signals with the appropriate transcription factor modules. In harmony with the Synthesis-Diffusion-Clearance model of gradient formation, interactions of morphogens with proteins in the extracellular space (secreted proteins, constituents of the extracellular matrix and extracellular parts of transmembrane proteins) play crucial roles in gradient formation and the graded activity of morphogens leads to region-specific transcriptional responses and cell fates [41]. The interplay of signaling by different morphogen gradients constitutes a complex developmental program that helps translate genetic information into three-dimensional patterns of cells. Development is hierarchical in as much as the initial steps specify major subdivisions of the body, followed by subdivision to organs, tissues, and different cell types [40].

Comparison of proteomes from different animal phyla and the closest unicellular relatives of animals has begun to shed light on the origins of the different morphogen signaling pathways and the various secreted, transmembrane and extracellular matrix proteins that are crucial for spatial patterning. These studies have revealed that although domain-constituents of the majority of these proteins were probably present in the unicellular ancestors of animals, the characteristic multidomain architectures of these proteins evolved only in Metazoa [1,2,10,42,43,44,45]. Thus, the major conclusion that emerged from comparative genomic studies is that the evolution of the different morphogenetic pathways and sophisticated cell–cell recognition systems essential for spatial patterning may not have required the evolution of novel domains, but rather the creation of novel multidomain proteins through domain shuffling.

It must be emphasized that multidomain proteins are unique in that a large number of functions may coexist in these molecules, making them indispensable constituents of regulatory or structural networks where multiple interactions are essential. We have shown that, as a corollary of their involvement in multiple interactions, formation of novel multidomain proteins contributed significantly to the evolution of increased organismic complexity [46]. Since Metazoa share a unique set of multidomain proteins involved in morphogen signaling, cell–cell adhesion, cell–cell recognition and cell–matrix interactions that are essential for multicellularity, we have suggested that the formation of these proteins through domain shuffling played a dominant role in the emergence of Metazoa [46,47,48,49,50,51].

Although it is now widely accepted that creation of these multidomain proteins was indispensable for the evolution of multicellularity, it has been suggested that changes in gene regulation might have been more critical for the evolution of Metazoa [52]. Based on the analysis of the genome of *Capsapora*, a close unicellular relative of animals, Sebé-Pedrós et al. [52] suggested that changes in the regulatory genome, “the appearance of developmental promoters and distal enhancer elements, rather than of gene innovations, may have been the critical events underlying the origin of multicellular organisms” [52]. These authors have proposed that “the unicellular ancestor of Metazoa already had a complex gene repertoire involved in multicellular functions” and “the emergence of animal multicellularity was linked to a major shift in genome cis-regulatory complexity, most notably the appearance of distal enhancer regulation” [52]. The implicit suggestion of this study is that the evolution of the gene repertoire involved in multicellular functions preceded the evolution of a complex regulatory genome and gene innovation was less critical for the emergence of animals than changes in gene regulation.

I disagree with this conclusion. In my opinion, no increase in the complexity of gene regulation networks could substitute for the absence of structural genes that encode proteins indispensable for cell–cell interactions critical for morphogenesis. In the next sections, I discuss evidence that suggests that gene innovation, the creation of novel multidomain proteins inextricably linked to spatial patterning, played a decisive role in the emergence of metazoan type multicellularity.

### 2.3. The Role of Introns and Exon Shuffling in the Evolution of Metazoa

#### 2.3.1. Introns and Domain Shuffling

Our studies on mutidomain proteins of animals in the early 1980s have led us to suggest that they evolved by domain shuffling [53,54]. Since the constituent domains of the multidomain proteins of blood coagulation and fibrinolysis were found to be separated by introns in their genes, we have suggested that intronic recombination facilitated the shuffling of these domains [54]. Analysis of the exon–intron structure of the genes of numerous mutidomain proteins of animals have also revealed that gene assembly by exon shuffling is reflected not only by a correlation between the domain organization of the proteins and exon–intron organization of their genes. The modules involved in domain shuffling were found to be ‘symmetrical’ in the sense that the introns flanking the domains were of the same phase, the shuffled modules, thus, have 3n bases [55]. The significance of this observation is that only modules that have introns of the same phase class at both their ends (symmetrical modules of classes 1–1, 2–2, and 0–0) can be shuffled by intronic recombination without disrupting the reading frame. Thus, a diagnostic sign of gene assembly by exon shuffling is a correlation between the domain organization of the proteins and exon–intron organization of their genes as well as the nonrandom phase usage of the introns that separate domains [55].

Analyses of the evolutionary distribution of proteins assembled from modules by exon shuffling have shown that they are restricted to the animal kingdom [47,48,49,50,51,55,56]. Although such proteins are present in all major groups of Metazoa from sponges to chordates, there is practically no evidence for modular proteins produced by exon shuffling in other groups of eukaryotes, leading us to suggest that exon shuffling became significant at the time of the appearance of the first multicellular animals [47,48,49,50,51,55,56]. In harmony with this conclusion, França et al. [57] have found that exon shuffling is significant throughout Metazoa (including *Trichoplax adhaerens* and *Nematostella vectensis*), whereas the choanoflagellate *Monosiga brevicollis* and the plant *Arabidopsis thaliana* show no evidence of exon shuffling [57].

Surveys of metazoan proteins assembled by exon shuffling have also revealed that they are mostly extracellular: secreted proteins, extracellular parts of transmembrane proteins [47,48,49,50,51,56]. This group includes proteins of the extracellular matrix, membrane-associated proteins mediating cell–cell and cell–matrix interactions, receptor proteins regulating cell–cell communications that are involved in controlling morphogenetic and differentiation processes, thus, determining the basic body plans of Metazoa [47,48,49,50,51]. Since the modular proteins created by exon shuffling are associated with and are essential for multicellularity, we have suggested that the rise of exon shuffling could contribute to metazoan radiation [47,48,49,50,51,56].

Our analyses have also revealed that the rich repertoire of extracellular and transmembrane proteins created by exon shuffling in the metazoan lineage was assembled from just a few dozen domain-types [47,48,49,50,51]. Surprisingly, although class 1–1, class 0–0, and class 2–2 modules are equally suitable for module shuffling [55], all the domain types used in exon shuffling were found to belong to class 1–1 [47,48,49,50,51]. In addition to the extracellular or membrane-associated modular proteins, there are some intracellular modular proteins that have also evolved by exon-shuffling of class 1-1 modules. For example, the muscle proteins twitchin, titin, projectin, myomesin/skelemin have been constructed from class 1–1 immunoglobulin and fibronectin type III modules by exon-shuffling within the metazoan lineage [51].

These observations on the role of introns in the evolution of metazoan proteins have posed several, interrelated questions. Why is exon shuffling restricted to Metazoa? What is the explanation for the observation that it contributed more significantly to extracellular than intracellular proteins? What is the explanation for the enigmatic preference of shuffling for class 1–1 modules? In the next sections I address these questions.

#### 2.3.2. Intron Evolution, Intron Invasions, Exon Shuffling and the Rise of Metazoa

Pre-mRNA introns characteristic of eukaryotic nuclear protein genes evolved from group II self-splicing introns, parallel with the evolution of a sophisticated splicing machinery. It has been suggested that the emergence of the eukaryotic cell involved a catastrophic intron invasion; the group II intron invasion was triggered by the establishment of the endosymbiosis of an archaeal host and an α-proteobacterium that contained a large number of invasive group II elements [58].

The distinction between self-splicing and spliceosomal pre-mRNA introns has important consequences for the significance of exon shuffling via recombination in introns [59]. Since self-splicing introns encode an essential function, the capacity to act as ribozymes, their sequence is not tolerant to intronic recombination that mediates exon shuffling. On the other hand, pre-mRNA introns that play only a minor role in their own excision, are tolerant to large deletions/insertions that accompany intronic recombination involving non-homologous introns. Accordingly, exon shuffling could become increasingly significant with the evolution and spread of spliceosomal introns that could accommodate large segments of middle repetitious sequences, increasing the chances of intronic recombination [50,59].

Our observation that exon shuffling became significant only in the metazoan lineage suggests that a population of introns appeared at the root of metazoan evolution that was particularly suitable for exon shuffling. It seems possible that the rise of exon shuffling was due to an invasion of the genome of the unicellular metazoan ancestor by a new breed of introns, capable of facilitating intronic recombination. There is indeed evidence that supports the assumption that intron invasion may have played a significant role in the unicellular-metazoan transition. Several studies indicate that, due to a burst of intron invasion during a limited time span at the origin of animals, the last common ancestor of Metazoa had the highest intron density among eukaryotes [58,60,61,62,63].

Our conclusions that exon-shuffling events are practically restricted to animal evolution and that the majority of multidomain proteins that have evolved via exon shuffling are essential for multicellularity also imply that the intron invasion played a crucial role in the unicellular-metazoan transition as it permitted the rise of exon shuffling [48,49,50,51].

Although this conclusion is now widely accepted, I must mention that there are other explanations as to why intron invasion could play a significant role in metazoan evolution. For example, Grau-Bové et al. [63] have suggested that intron gains could have a major impact on evolution of organismic complexity of animals since, due to alternative splicing, more intron-dense genomes tend to encode more complex transcriptomes and proteomes [63]. Although these two explanations for the evolutionary significance of intron invasion are not mutually exclusive, its role in exon shuffling appears to be more decisive as it contributed specifically to the creation of the toolkit that is essential for spatial patterning in Metazoa.

In a more recent paper, Grau-Bové et al. [64] have extended their studies on the significance of alternative splicing in animal evolution. In harmony with earlier conclusions [65,66], the authors have found that animals have higher rates of exon skipping than other eukaryotes, including plants. Significantly, the authors have found that exon skipping is significantly enriched for alternative splicing events that preserve the reading frame [64]. Bilateria and Cnidaria have a high fraction of 3n exons among their exon skipping events, but no such enrichment is observed in plants or other eukaryotes, but the authors did not provide an explanation why the pressure to maintain reading frame during alternative splicing might be a trait specific for Bilateria and Cnidaria [64]. We have suggested that the higher frequency of frame-preserving exon skipping in Metazoa than in all other eukaryotes reflects the fact that the majority of multidomain proteins unique to Metazoa were assembled by exon shuffling from ‘symmetrical’ 3n modules, that are particularly well suited to exon-skipping [66,67].

Very little is known about molecular characteristics of the introns that invaded the genome of the premetazoan ancestor or the actual mechanism of their insertion. Several types of mechanisms may contribute to the insertion of introns into genes, such as transposition, transposon insertion, tandem genomic duplication, intron gain during Double-Strand Break Repair, insertion of a group II intron, intron transfer, and intronization [68]. Nevertheless, it is widely accepted that intron transposition that may occur via a process similar to group II intron retrotransposition played a significant role in shaping the exon intron structures of eukaryotes [69,70,71,72,73]. Recent evidence suggests that massive transposition of introns had a major impact on the intron landscape of *Micromonas*, a unicellular green alga, suggesting that intron gain by transposition may be more widespread than previously thought [74].

According to the transposition hypothesis of intron gain, the spliceosomal components may remain associated with a recently excised intron and then attach at a potential splice site of a mRNA, where they catalyze the reverse reaction, modifying it by inserting the excised intron. The modified mRNA is reverse transcribed and the resulting cDNA participates in a recombination with its parent gene, inserting a novel intron into the gene. The most attractive feature of this mechanism is that it ensures that the inserted sequence has the full complement of intron signature sequences (donor, acceptor, branch point sequences) required for efficient splicing and that reverse splicing guides intron insertion to sites—proto-splice sites—that possess the appropriate exonic features that facilitate efficient splicing [75]. Significantly, selection favors insertion of perfect spliceosomal introns into proto-splice sites since the inserted intron will be accepted only if it is efficiently spliced out. Intron insertion at a site that does not conform to the proto-splice-site consensus will not be tolerated since the retained intron would disrupt the structure of the affected protein. In harmony with these expectations, there is evidence that insertion of novel introns is preferred at sites that conform to the AG/G exon splice site consensus sequence [76]. The work of Coghlan and Wolfe [77] is especially instructive. These authors, comparing the genomes of the nematodes *Caenorhabditis elegans* and *Caenorhabditis briggsae*, have identified numerous cases where a difference between the exon–intron structure of equivalent genes of *C. elegans* and *C. briggsae* was caused by intron gain. Significantly, such novel introns were found to have a much stronger exon splice site consensus sequence (AG/G) than the general population of introns, suggesting that novel introns tend to be inserted at AG/G sites.

In summary, the most plausible hypothesis for the nature of the massive intron invasion at the origin of Metazoa is that it occurred through transposition, preferentially to sites (AG/G proto-splice sites) that were optimal for the efficient removal of the intron invader by the spliceosome [51]. This hypothesis implies that, at the origin of Metazoa, some changes occurred in components of the intron-spliceosome complex that increased the chances of reverse splicing and, thus, favored massive intron gain.

#### 2.3.3. The Mysterious Predominance of Class 1–1 Modules

As mentioned above, the domain types used for the construction of proteins essential for multicellularity of animals all belong to class 1–1. Rogozin et al. [58,60] have pointed out that this observation is even more intriguing as this strong preference for phase 1 introns is in sharp contrast with the observation that there is a general excess of phase 0 introns among the three intron-phase classes [58,60]. The mysterious predominance of class 1–1 modules has led these authors to conclude, that “evolution of the introns flanking mobile domains is fundamentally different from the evolution of introns in conserved portions of genes, but the nature of these differences remains to be understood” [58,60].

In our earlier work, we have suggested that a fundamental difference between the introns flanking the mobile domains and other introns is that the former belong to the group of introns that were inserted when introns invaded the premetazoan genome [48,56]. These introns were the ones that actually played the most decisive role in the rise of exon shuffling and the evolution of multicellularity. In our ‘modularization hypothesis’, we have suggested that the mobile domains suitable for exon shuffling arose from non-mobile domains by insertion of introns at their boundaries [48,56].

However, it remained to be explained why class 1–1 module types are much more numerous than class 0–0 and class 2–2 modules. According to the modularization hypothesis, the question of this enigmatic preference for class 1–1 modules could be rephrased: why would domains have a greater chance of modularization as class 1–1 rather than as class 2–2 or class 0–0 modules? Why would intron insertion be preferred at the boundaries of a domain in phase 1?

The answer to these questions lies in the fact that in protein-coding regions proto-splice sites are translated, therefore, the base preferences of exon–intron junctions are also manifested in amino acid preferences. If the intron inserted into the AG/G proto-splice site is a phase 2 intron then it splits an AGG (Arg) codon of the mRNA. By a similar reasoning, if the AG/G site is split by a phase 1 intron then it splits one of the four codons for glycine (GGN), whereas for phase 0 introns there is a great freedom of choice of codons flanking the intron. As a corollary, biased amino acid composition of protein segments is reflected in the abundance or scarcity of proto-splice sites and in a biased phase distribution of proto-splice sites [48,49,50,51,56]. In general, linker regions connecting protein domains have a biased amino acid composition where glycine is overrepresented therefore it might be expected that in these regions introns are most likely to be inserted in phase 1 [48,49,50,51,56].

There is an additional source of bias favoring the creation of class 1–1 modules. The vast majority of class 1–1 modules is extracellular that originated from single-domain extracellular proteins [48,49,50,51,56]. Since extracellular proteins possess a secretory signal peptide, one of the introns initiating modularization had to be inserted at the boundary separating the signal-peptide domain from the protein domain [48,49,50,51,56]. The amino acid sequence at this boundary is far from random as the cleavage site recognized by the signal peptidase is enriched in small neutral residues such as glycine therefore intron insertion is expected to be preferred in phase 1. Analysis of the exon–intron structures of thousands of human genes has indeed revealed that there is a statistically highly significant excess of phase 1 introns in the vicinity of the signal peptide cleavage sites of secreted proteins [78]. In other words, since modularization of domains of secreted proteins is most likely to create class 1–1 modules, the predominance of such modules may simply reflect the fact that they were produced from proteins possessing a secretory signal-peptide.

In harmony with the role of class 1–1 modules in metazoan evolution, a systematic analysis of animal genomes from nematodes to mammals revealed that domains flanked by phase 1 introns are much more abundant than those flanked by phase 0 or phase 2 introns [79]. Although targeting of introns to proto-splice sites may have favored the creation of class 1–1 modules from extracellular domains, it is likely that the abundance of class 1–1 modules in Metazoa reflects the action of additional selection forces.

The frequency of the use of a given domain-type to build novel multidomain proteins depends on the selective value of the resulting protein. Stability and folding autonomy of domains may be of utmost importance for the viability of the multidomain proteins, it seems, thus, likely that the most widely used domains have been selected according to the rate, robustness, and autonomy of folding [51,80]. It is noteworthy in this respect, that class 1–1 modules used most frequently in the construction of multidomain proteins are not random representatives of the protein fold universe: they belong to the mainly β class of proteins, with only a negligible fraction being assigned to the mainly α class of protein folds [51,81]. The fact that the structural distribution of extracellular domains is in general shifted toward the mainly β proteins [82] suggests that such domains are more stable in the extracellular environment than other fold classes.

Functional aspects must have also contributed to the proliferation of certain domains through selective retention of functionally relevant novel multidomain architectures. It seems very likely that, with the emphasis shifting from cellular differentiation to spatial patterning, there was a great demand for extracellular domains that can serve to build constituents of the extracellular matrix, extracellular parts of transmembrane proteins that mediate cell adhesion, cell-recognition, cell–matrix interactions, and reception of morphogen signals. It seems, thus, very likely that the preponderance of extracellular class 1–1 modules in Metazoa is in a great part due positive selection for proteins that were crucial for the temporal to spatial transition.

Intriguingly, although domain shuffling played a significant role in the evolution of proteins involved in transcription factor modules [28,37], there is no evidence for a role of exon shuffling in these processes. In our view, the most plausible explanation for this difference between the multidomain proteins of the intracellular and intercellular toolkits of metazoan multicellularity is that the evolution of the proteins of the transcription factor modules preceded the intron invasion that triggered the rise of exon shuffling and the creation of the proteins controlling spatial patterning in Metazoa.

Note that, according to this interpretation, the temporal correlation between the rise of exon-shuffling and metazoan radiation is not just a coincidence. The availability of this evolutionary mechanism could actually contribute significantly to metazoan evolution, by creating the repertoire of genes indispensable for spatial patterning. The prominent role of a limited set of class 1–1 modules and exon shuffling in the evolution of multidomain proteins unique to Metazoa highlights the fact that evolution is an opportunistic process: the preexisting constitution of the organism determines in what direction it will go.

## 3. Conclusions

The genetic toolkit of multicellularity of Metazoa consists of two major components: genetic factors controlling differentiation of cell types and those involved in the establishment of the spatial pattern of different cell types. We have shown that extracellular and transmembrane proteins crucial for spatial patterning of different cell types have been assembled by exon shuffling, but there is no evidence for a similar role of exon shuffling in the evolution of proteins of transcription factor modules that control differentiation of cell types.

**Figure 1:**
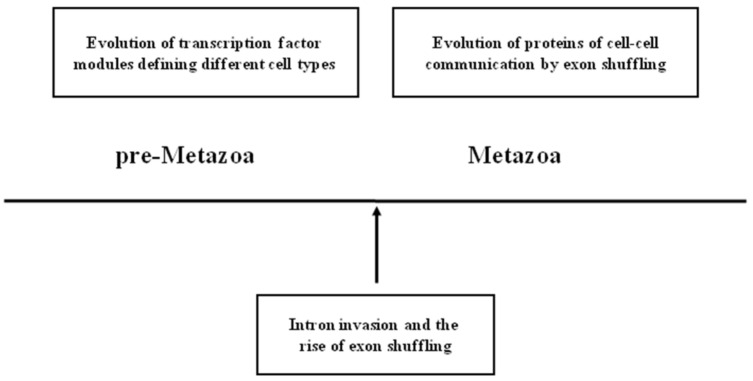
Schematic representation of the relative timing of the key events leading to the emergence of metazoan-type multicellularity. The figure emphasizes that the evolution of transcription factor modules defining different cell types of unicellular and colonial pre-Metazoa preceded the intron invasion that led to the rise of exon shuffling and the creation of cell–cell communication proteins that are essential for complex multicellularity of Metazoa.

In harmony with the temporal-to-spatial transition hypothesis, we suggest that the explanation for this difference is that the evolution of the transcription factor modules that define cells types preceded the burst of intron invasion that resulted in a rise of exon shuffling and the formation of genes essential for spatial patterning (Figure 1).

## Data Availability

Not applicable.
